# Immunomodulatory properties of *Brucella melitensis* lipopolysaccharide determinants on mouse dendritic cells *in vitro* and *in vivo*

**DOI:** 10.1080/21505594.2017.1386831

**Published:** 2017-10-02

**Authors:** Yun Zhao, Sean Hanniffy, Vilma Arce-Gorvel, Raquel Conde-Alvarez, SangKon Oh, Ignacio Moriyón, Sylvie Mémet, Jean-Pierre Gorvel

**Affiliations:** a Centre d'Immunologie de Marseille-Luminy, CIML, Aix Marseille Univ, CNRS, INSERM, Marseille, France; b Departamento de Microbiología y Parasitología, Instituto de Salud Tropical, Instituto de Investigación Sanitaria de Navarra, Universidad de Navarra, c/Irunlarrea 1, Pamplona, Spain; c Baylor Institute for Immunology Research, 3434 Live Oak St., Dallas, TX, U.S.A

**Keywords:** *Brucella*, dendritic cell, intracellular bacteria, lipopolysaccharide, T cell proliferation, toll-like receptor, vaccine

## Abstract

The lipopolysaccharide (LPS) is a major virulence factor of *Brucella*, a facultative intracellular pathogenic Gram-negative bacterium. *Brucella* LPS exhibits a low toxicity and its atypical structure was postulated to delay the host immune response, favouring the establishment of chronic disease. Here we carried out an in-depth *in vitro* and *in vivo* characterisation of the immunomodulatory effects of *Brucella* LPS on different dendritic cell (DC) subpopulations. By using LPSs from bacteria that share some of *Brucella* LPS structural features, we demonstrated that the core component of *B. melitensis* wild-type (Bm-wt) LPS accounts for the low activation potential of *Brucella* LPS in mouse GM-CSF-derived (GM-) DCs. Contrary to the accepted dogma considering *Brucella* LPS a poor TLR4 agonist and DC activator, Bm-wt LPS selectively induced expression of surface activation markers and cytokine secretion from Flt3-Ligand-derived (FL-) DCs in a TLR4-dependent manner. It also primed *in vitro* T cell proliferation by FL-DCs. In contrast, modified LPS with a defective core purified from *Brucella* carrying a mutated *wadC* gene (Bm-*wadC*), efficiently potentiated mouse and human DC activation and T cell proliferation *in vitro*. *In vivo*, Bm-wt LPS promoted scant activation of splenic DC subsets and limited recruitment of monocyte- DC like cells in the spleen, conversely to Bm-*wadC* LPS. Bm-*wadC* live bacteria drove high cytokine secretion levels in sera of infected mice. Altogether, these results illustrate the immunomodulatory properties of *Brucella* LPS and the enhanced DC activation ability of the *wadC* mutation with potential for vaccine development targeting *Brucella* core LPS structure.

## Introduction

The Gram-negative bacteria of the genus *Brucella* are facultative intracellular pathogens that cause brucellosis, a worldwide zoonosis affecting wild life and livestocks with serious economic losses.^1^ This disease is transmitted to humans via contaminated food or aerosols and leads to chronic inflammation. Human brucellosis is difficult to treat with antibiotics and no effective vaccine exists yet to prevent it. *Brucella* is characterized by its ability to escape early detection by the innate immune system. An efficient strategy *Brucella* has developed to achieve such stealthy behaviour is to modulate pathogen-associated molecular pattern (PAMP) to avoid recognition by pathogen recognition receptors (PRR) on host cells like macrophages or dendritic cells (DCs).^2–5^

The most conspicuous PAMP bearing component of the surface of Gram-negative bacteria is the LPS. This molecule is composed of a hydrophobic lipid A, embedded in the outer membrane, linked to a charged oligosaccharide core associated with a hydrophilic O-polysaccharide chain (O-chain) that covers the bacterial surface.^6^ Canonical LPS, like *Escherichia coli* (*E. coli*) LPS, expresses a lipid A made of a glucosamine disaccharide linked predominantly to C12 to C14 acyl chains in ester, amide and acyl-oxyacyl bonds, which are recognised by the toll-like receptor 4 (TLR4)/MD-2 complex. In contrast, *Brucella* lipid A is a 2,3-diaminoglucose disaccharide substituted with C16, C18, C28 and other very long acyl chains. This peculiar structure is a poor agonist of TLR4/MD-2 and therefore a paradigm has emerged proposing *Brucella* LPS as a crucial virulence factor that hampers recognition by PRRs and plays essential roles during infection.^2,4,5,7^

DCs are the most potent antigen-presenting cells, equipped with a variety of PRRs, which detect bacterial PAMPs and trigger downstream signalling, resulting in antigen uptake and processing as well as cytokine secretion. These cells are regarded as ‘sentinels against pathogens’ for induction of T-dependent adaptive immunity.^8^ A variety of DC subsets exist *in vivo* as distinguished by specific cell surface markers and functions.^9–11^ Under steady-state conditions in mice, at least three splenic DC subsets have been identified: bone marrow stromal antigen-2 (Bst-2)^+^ plasmacytoid DCs (pDCs), CD8α^+^ and CD11b^+^ conventional DCs (cDCs).^8,10^ These subsets of DCs can display shared as well as distinct functions in controlling host immune responses and this is partly due to the expression of PRRs. As such, pDCs predominantly display TLR7 and 9, and sense viral and bacterial pathogens releasing high levels of Type I interferons (IFN-I).^12^ CD8α^+^cDCs express TLR3, 4 and 9, and play a critical role in the induction of cross-presentation *in vivo*.^10,13^ CD11b^+^ cDCs exhibit TLR4, 7 and 9, and present a weaker cross-priming ability. This latter subset, mainly localised in the marginal zone of the spleen at the steady state, migrates upon stimulation to the T cell areas and secretes cytokines.^10,13^ Upon microbial stimulation *in vivo*, monocyte-derived DC (MO-DC) are also differentiated and recruited from blood monocytes to immune T cell areas.^14^ mo-DCs also have the capacity to activate antigen-specific CD4^+^ T cell responses and to cross-prime CD8^+^ T cells, during viral, bacterial, and parasitic infections.^15^ They are distinguished from conventional DCs by the expression of CD64 and MAR-1 at least in the lung and mesenteric lymph nodes^16,17^ and DC-SIGN.^18^

*In vitro* generated mouse granulocyte macrophage-colony stimulated factor (GM-CSF)- or FMS-like tyrosine 3 ligand (Flt3L)-derived bone marrow DCs (GM-DCs and FL-DCs, respectively) are widely used to decipher the immunology and cell biology of DCs. Flt3L treatment of murine bone marrow progenitors generates three distinct DC subtypes including B220^+^pDCs, CD24^+^cDCs and CD11b^+^cDCs, which were proven to be equivalent of splenic pDCs, CD8α^+^cDCs and CD11b^+^cDCs, respectively.^10,11^ GM-DCs consist of a DC population suggested to be the equivalent to the mo-DCs that emerge during inflammation *in vivo* and of CD11c^+^MHCII^+^ macrophages.^19–21^

We previously demonstrated that LPSs with a partially defective core, which were purified from *Brucella* mutants with a mannosyltransferase (*wadC*) gene deletion, were much more potent than *Brucella* wild-type LPS at activating mouse BMDCs, as measured by assessing DC phenotype maturation and and secretion of pro-inflammatory cytokines such as IL6 and TNFα.^22,23^ However, these studies were based on the sole analysis of GM-DCs. Given that the magnitude and profile of DC activation triggered by *Brucella* infection i*n vitro* vary according to the diversity of DC subtypes and functions,^24^ we carried out further characterisation of the immunomodulatory properties of *B. melitensis* LPS (Bm-wt and Bm-*wadC* LPS) in different DC subsets *in vitro* and *in vivo*. *In vitro*, we found that Bm-wt LPS activated FL-DCs unlike GM-DCs. By using LPSs from various sources that share some of *Brucella* structural features, we demonstrated that the core component of Bm-wt LPS was responsible for the low activation potential of *Brucella* LPS in mouse GM-DCs and human GM/IL-4 monocyte-derived DCs. Even though effective, Bm-wt LPS activation of FL-DCs was not powerful enough to lead to full T cell proliferation. In contrast, Bm-*wadC* LPS succeeded in activating both GM-DCs and FL-DCs, consequently accelerating CD4^+^ and CD8^+^ T cell proliferation. *In vivo*, the *wadC* mutation conferred an enhanced activation ability to *Brucella* LPS in splenic conventional DC subsets and elicited efficient mo-DC like cell mobilisation. This improved immunogenic ability of Bm-*wadC* LPS was reflected by potent secretion of Th1 cytokines in serum of mice infected by the *Brucella wadC* mutant.

## Results

### The core structure of Brucella LPS inhibits GM-DC activation

**Figure 1.**
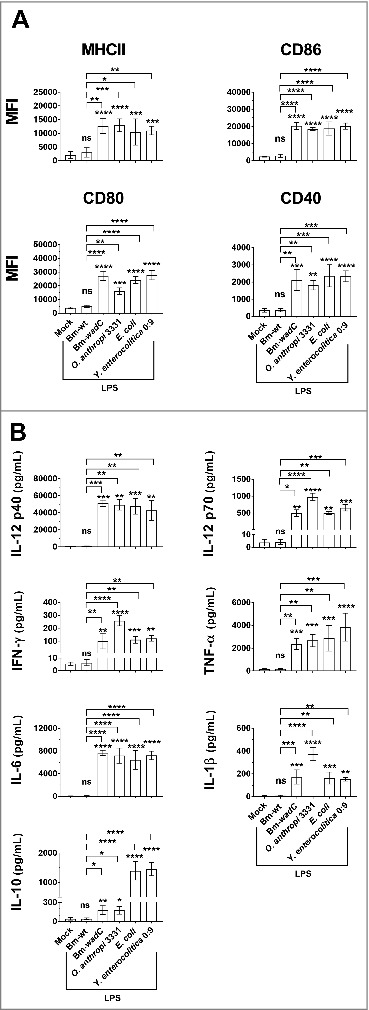
Comparison of several LPSs carrying different structure moieties in GM-DCs revealed a shield function of the *Brucella* core component. GM-DCs were non-treated (Mock) or stimulated with Bm-wt LPS, Bm-*wad*C LPS, *Ochrobactrum anthropi* 331 LPS, *Yersinia enterocolitica* O:9 LPS or *E. coli* LPS for 24 h. *Brucella* LPSs, *Y*. *enterocolitica* and *O*. *anthropi* LPS were used at the concentration of 10 μg/mL and *E*. *coli* LPS was at 100 ng/mL. **(A)** MHCII and co-stimulatory molecule levels of expression (MFI, Mean of Fluorescence Intensity) were measured by flow cytometry. **(B)** Cytokine secretion was determined in culture supernatants by ELISA. The graphs show combined data from at least three independent experiments. All error bars are standard deviations obtained from pooled data. Significant differences from mock or from Bm-wt LPS are shown. *, *P* < 0.05; **, *P* < 0.001; ***, *P* < 0.0001; ****, *P* < 0.00001. ns, non-significant.

To unravel the molecular determinants accounting for the lack of GM-DC activation by *Brucella* wild-type (wt) LPSs,^22,23^ we first assessed the role of the different sugar moieties of Bm LPS on DC activation *in vitro* by comparing the effect of LPSs from various sources that share some of *Brucella* structural features. As mentioned above, Bm-*wadC* LPS has a partially defective oligosaccharide core but displays intact O-polysaccharide and lipid A compared to Bm-wt LPS.^22,23,25^ More precisely, the defect in the Bm-*wadC* LPS corresponds to a loss of an oligosaccharide branching connected to the core section linking lipid A and the O-polysaccharide.^23,25^ *Yersinia enterocolitica* O:9 LPS exhibits the same O-chain homopolymer as Bm-wt LPS but its lipid A is alike *E. coli* LPS.^26,27^ *Ochrobactrum anthropi* 3331 LPS carries a “*Brucella*-type*”* lipid A but different core and O-chain sugars.^26–28^ *E. coli* LPS differs by its overall structure from that of Bm-wt LPS, but as aforementioned has in common lipid A and core features with *Y. enterocolitica* O:9 LPS^26,27^ **(Fig. S1**). After 5 days of culture, murine bone marrow GM-DCs were stimulated for 24 h by this array of LPSs, all at 10 µg/mL but *E. coli* LPS at 100 ng/mL as described previously.^22^ Non-adherent cells were used for phenotypic analysis by flow cytometry and cell culture supernatants for cytokine measurement by ELISA. Confirming prior reports, Bm-wt LPS did not activate GM-DCs in terms of surface expression of activation markers (Fig. 1A) and secretion of inflammatory cytokines (Fig. 1B). In GM-DCs, all other LPSs drove induction of expression of surface activation markers, including MHCII, CD80, CD86 and CD40, at comparable levels (Fig. 1A). Similar cytokine (IL-12p40, IL-12p70, IFNγ, TNFα, IL-6, IL-1β) secretion patterns were also generated by all LPSs with the exception of Bm-wt LPS (Fig. 1B). Remarkably as regards IL-10, three sets of LPSs were distinguished: the inactive Bm-wt LPS, the Bm-*wadC* and *O. anthropi* 3331 LPSs, which share a “*Brucella* type”-lipid A and led to intermediate secretion levels and the *E. coli* LPS and *Y. enterocolitica* O:9 LPSs, which bear the same “*E. coli* type-lipid A” and elicited 5 times more secreted IL-10 levels. Altogether, the fact that similar DC activation patterns were achieved by a “*Brucella*-type*”* lipid A (Bm-*wadC* and *O. anthropi* 3331) or O-chain (Bm-*wadC* and *Y. enterocolitica* O:9) LPSs revealed that these molecular structures without the inhibitory effects of *Brucella* WT core component have the ability to trigger GM-DC activation. These results also indicated that the outer core deficiency by itself considerably increased DC activation *in vitro*.

### WadC Brucella LPS activates human mo-DCs

**Figure 2.**
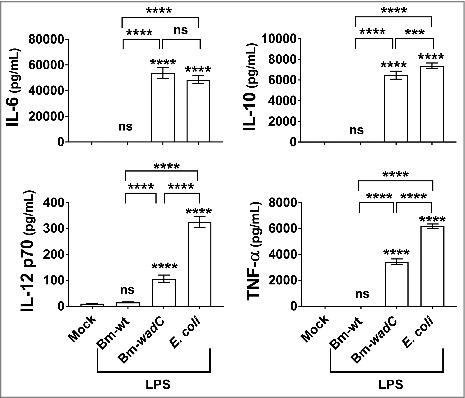
Unlike Bm-wt LPS, Bm-*wadC* LPS elicited cytokine secretion from human mo-DCs. Human GM-CSF and IL-4 derived mo-DCs were non-treated (Mock) or stimulated with Bm-wt LPS, Bm-*wadC* LPS or *E. coli* LPS for 72 h. All LPSs were used at a concentration of 20 ng/mL. Cytokine secretion was determined in culture supernatants by ELISA. The graphs show combined data from at least four independent experiments with n = 1 animal per condition. All error bars are standard deviations obtained from pooled data. Statistical analysis was performed with the parametric one-way ANOVA test, followed by variance analysis with the Tukey and Dunnett test. Significant differences from mock or from Bm-wt LPS were identical. ***, *P* < 0.0001; ****, *P* < 0.00001. ns, non-significant.

We then determined whether *Brucella* LPSs would elicit activation of DCs from human origin *in vitro*. Human monocytes DCs (mo-DCs) were derived from PBMC from healthy blood donors in the presence of GM-CSF and IL-4 (GM/IL-4 mo-DCs). After 7 days of culture, these mo-DCs were stimulated for 72 h with Bm-wt or Bm-*wadC* LPS, both at 20 ng/mL. Cell culture supernatants were used for inflammatory cytokine measurement by ELISA. We found that Bm-wt LPS in human GM/IL-4 mo-DCs did not trigger any secretion of IL-6, IL-12p70, TNFα or IL-10. In contrast, Bm-*wadC* LPS prompted strong secretion of IL-12p70 and TNFα while IL-6 and IL-10 secreted levels reached higher values, comparable to those obtained with *E. coli* (Fig. 2). These findings reminiscent of those observed with the GM-DC model reveal that our murine GM-DC data can be translated to human GM/IL-4 mo-DCs.

### Brucella LPS activates FL-DCs

**Figure 3.**
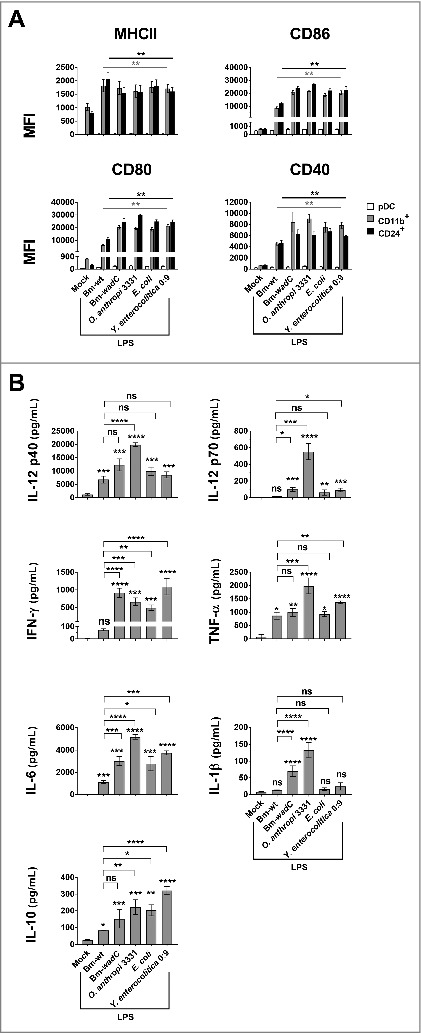
Bm-wt LPS preferentially triggered maturation of FL-DCs *in vitro*, while Bm-*wadC* LPS activated that of both GM-DC and FL-DC subsets. FL-DCs were non-treated (Mock) or stimulated for 24 h with Bm-wt LPS, Bm-wadC LPS, *Ochrobactrum anthropi* 331 LPS, *Yersinia enterocolitica* O:9 LPS or *E. coli* LPS. *Brucella* LPSs, *Y. enterocolitica* and *O. anthropi* LPS were used at the concentration of 10 μg/mL and *E. coli* LPS was at 100 ng/mL. **(A)** MHCII and co-stimulatory molecule expression levels (MFI, Mean of Fluorescence Intensity) were measured by flow cytometry. (**B**) Cytokine secretion was determined in whole FL-DC culture supernatants by ELISA. The graphs show combined data from at least three independent experiments. All error bars are standard deviations obtained from pooled data. Significant differences from mock only (**A**) or from mock or from Bm-wt LPS (**B**) are shown. *, *P* < 0.05; **, *P* < 0.001; ***, *P* < 0.0001; ****, *P* < 0.00001. ns, non-significant.

We next examined whether *Brucella* LPSs could activate FL-DCs. FL-DCs were generated from mouse bone marrow in the presence of the FLT3 Ligand (FL-DCs). After nine days of culture, these cells differentiated into heterogeneous DCs that could be split into 3 distinct populations, pDCs, CD24^+^ cDCs and CD11b^+^ cDCs (**Fig. S2**). Stimulations of FL-DC subsets were performed by culturing them for 24 h in the presence of Bm-wt or Bm-*wadC* LPS, *O. anthropi* 3331 LPS, *Y. enterocolitica* O:9 LPS (all at 10 µg/mL), or *E. coli* LPS at 100 ng/mL. Non-adherent cells were used for assessing surface phenotypes by flow cytometry and culture supernatants for inflammatory cytokine measurement by ELISA. Unlike GM-DCs (Fig. 1A), two of the FL-DC subsets (CD24^+^ cDCs and CD11b^+^ cDCs) upregulated MHC II, CD86, CD80, and CD40 expression on their surface in response to Bm-wt LPS. We further found that MHCII levels were alike for all LPSs, while expression of the other costimulatory molecules (CD86, CD80 and CD40) was enhanced but at lower levels than those driven by the other LPSs (Fig. 3A). Whereas pDCs were poorly activated by the different LPSs (Fig. 3A), Bm-wt LPS induced FL-derived CD24^+^ and CD11b^+^ cDCs to secrete significant amounts of certain cytokines, including TNF-α, IL-12p40, IL-6 and L-10. Others, such as IFN-γ, IL-12p70, IL-1β, were not significantly increased (Fig. 3B). In contrast, Bm-*wadC* LPS elicited a high activation profile, roughly comparable to that triggered by *E. coli* LPS in FL-DCs except for IL-10 and IL-1β (Fig. 3B). Interestingly, *Y. enterocolitica* O:9 LPS induced the greatest amount of IL-10 secretion from FL-DCs. In addition, *O. anthropi* 3331 was more potent than other LPSs at inducing IL-12p40, IL-12p70, TNF-α, IL-6, and IL-1β secretion from FL-DCs, revealing a specifc role for *O. anthropi* 3331 core and oligosaccharide moieties in these cells. Taken together, these findings disclosed that *Brucella* LPS exerted different modulatory properties depending on the targeted DC subtypes *in vitro*. They also unveil specific cytokine responses to the various LPS studied according to the DC model used.

### Bm-wadC LPS elicits T cell proliferation by both GM- & FL-DCs

**Figure 4.**
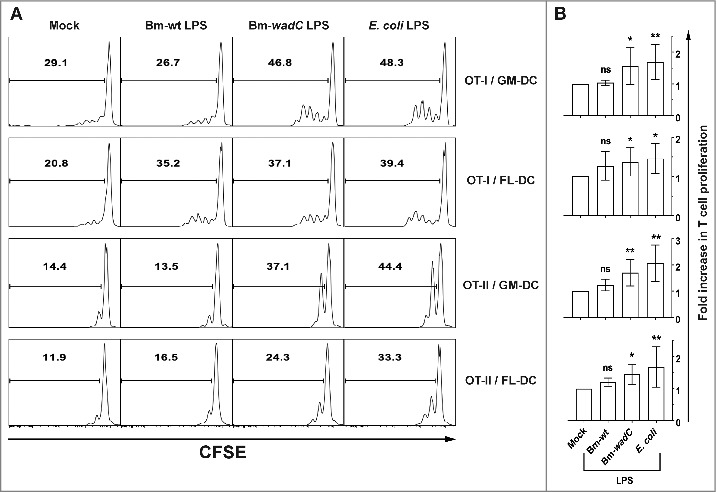
Bm-wt LPS-treated FL-DCs primed little T cell proliferation *in vitro*, while Bm-*wad*C LPS elicited significant T cell proliferation by both GM- & FL-DCs. GM- or FL-DCs were incubated for 16 h with OVA and were non-treated (Mock) or treated with different Bm LPSs (10 μg/mL) or *E. coli* LPS (100 ng/mL) for 8 h. Stimulated DCs were co-cultured with CFSE-labelled T cells from OT-I or OT-II mice. Proliferation of T cells was assessed after 2 days of co-culture by CFSE incorporation and flow cytometry. **(A)** One representative experiment is shown out of 3 independent experiments for OT-I and out of 5 for OT-II. **(B)** Pooled data are presented as mean ± standard deviation. Significant differences from mock. Significant differences from mock or from Bm-wt LPS were identical. *, *P*< 0.05; **, *P* < 0.001. ns, non-significant.

We further investigated whether the observed phenotypic maturation and cytokine release triggered by Bm-wt LPS and Bm-*wadC* LPS led to the enhancement of antigen-specific T cell responses. For this purpose, we performed DC-T cell co-cultures using either GM-DCs or FL-DCs together with carboxyfluorescein succinimidyl ester (CFSE)-labelled T cells from transgenic mice that express either a TCR specific for MHC class-I restricted ovalbumin (OVA) (OT-I) or a TCR specific for MHC class-II restricted OVA (OT-II). DCs incubated with ovalbumin were activated by different LPSs (Bm-wt or Bm-*wadC* LPS, both at 10 µg/mL), or *E. coli* LPS at 100 ng/mL, as a positive control) and co-cultured with freshly isolated OT-I (CD8^+^) and OT-II (CD4^+^) T cells for 48 h. Proliferation of T cells was traced by measuring CFSE dilution with flow cytometry. BMDCs incubated with LPS alone without OVA did not induce T cell proliferation (data not shown). Data from one representative experiment are shown in Fig. 4A and statistical analyses from pooled data of at least 3 independent experiments are presented in Fig. 4B. Bm-wt LPS generated a slight but non-significant increase of CD4^+^ or CD8^+^ T cell proliferation in FL-DCs only, ranging from 11.9% in mock-treated cells to 16.5% and 20.8% to 35.2%, respectively (Fig. 4). The two DC models treated with Bm-*wadC* LPS were potent activators of both CD4^+^ and CD8^+^ T cell proliferation, as was *E. coli* LPS. These results indicated that wt *Brucella* LPS despite FL-DC activation capacity was unable to trigger full T cell activation *in vitro*, whereas the Bm-*wadC* LPS, was responsible for a strong T cell activation in both GM- and FL-DCs.

### Emergence of a TLR4-mediated LPS-specific CD11c^int^CD11b^+^Bst-2^hi^ mo-DC-like cell subset in vivo

**Figure 5.**
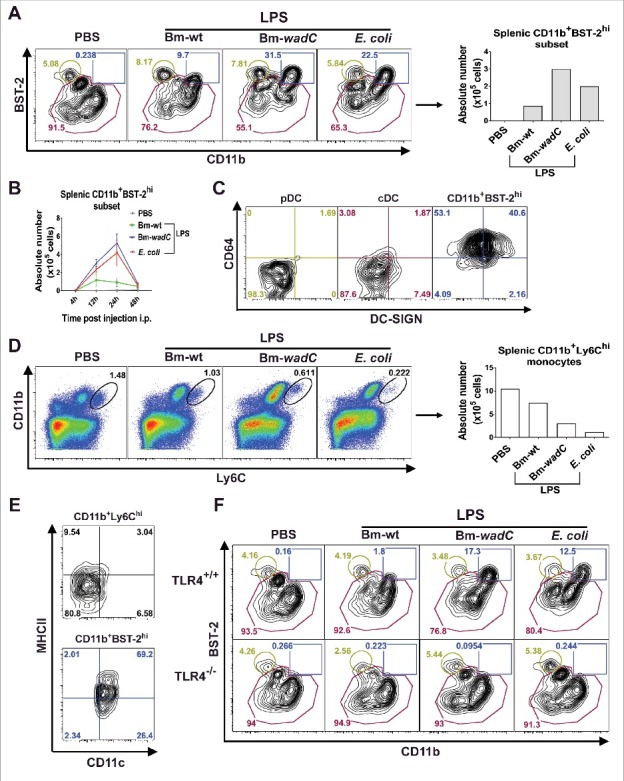
*In vivo*, LPSs favoured mobilisation of a mo-DC like cell type in a TLR4-dependent fashion. 6–8 weeks-old C57BL/6J mice were injected intraperitoneally with 1xPBS (PBS), Bm-wt LPS (30 μg/mouse), Bm-*wad*C LPS (20 μg/mouse) or *E. coli* LPS (10 μg/mouse). Quantities of injected LPS, calculated by KDO weight ratio, ensured equal molar weight for each of them. 4, 12, 24 and 48 h post-injection, mice were sacrificed, single-splenocyte suspensions were prepared and analysed by flow cytometry. **(A)** Dot plots of CD11b and BST-2 staining and absolute numbers of the CD11b^+^BST-2^hi^ subset 12 h post-injection. **(B)** Absolute numbers of the CD11b^+^BST-2^hi^ subset in spleen from injected mice at indicated time-points. **(C)** Dot plots of DC-SIGN and CD64 staining of the pDC, cDC and CD11b^+^BST-2^hi^ subpopulations 12 h post-injection. **(D)** Dot plots of Ly6C and CD11b staining and absolute numbers of the CD11b^+^Ly6C^hi^ monocytes 12 h post-injection. **(E)** Dot plots of CD11c and MHCII staining of the CD11b^+^Ly6C^hi^ and CD11b^+^BST-2^hi^ subpopulations 12 h post-injection. Data displayed from (**A** to **E**) are representative of 3 independent experiments, n = 3 mice per condition in each experiment. **(F)** 6–8 weeks-old TLR4^+/+^ or TLR4^−/−^ mice were injected with PBS, Bm-wt LPS, Bm-*wadC* LPS or *E. coli* LPS as described above. 12 h later, mice were sacrificed, single-splenocyte suspensions were prepared and total CD11c^+^ cells were analysed by flow cytometry. Dot plots of BST-2 and CD11b staining is representative of 3 independent experiments, n = 3 mice per condition in each experiment.

To determine if *Brucella* LPS exhibited immunomodulatory effects on DC subsets *in vivo*, we injected intraperitoneally Bm-wt LPS, Bm-*wadC* LPS or *E. coli* LPS into mice. Single-cell suspensions were prepared after enzymatic digestion and gentle dissociation of the spleens. After excluding neutrophils, NK cells, B cells, and T cells, the remaining cells were analysed by flow cytometry for CD11c expression. DCs were divided into CD11c**^h^**^i^ DCs and CD11c^int^ populations based on their CD11c expression level. CD11c^hi^ DCs were identified as conventional DCs (cDCs) due to the absence of BST-2^hi^ population. Under steady-state conditions, CD11c^int^ DCs were identified as pDCs (CD11b^lo^Bst-2^hi^) and cDCs (CD11b^lo to hi^ BST-2^lo to int^). Both cDC populations from CD11c^hi^ DCs and CD11c^int^ DCs were subdivided into CD8α^+^ and CD11b^+^ subsets based on CD11b and CD8α expression **(Fig. S3**). Fig. 5A shows that, upon stimulation with all LPSs, an additional population displaying a CD11b^+^BST-2^hi^ phenotype emerged within the CD11c^int^ DC population compared to splenic cells from PBS-injected animals. Accumulation of such population exhibited an identical kinetics between Bm-*wadC* LPS and *E. coli* LPS, culminating at 24 h and vanishing at 48 h. Of note, Bm-wt LPS presented a weaker effect with lower cell numbers and a peak that started to diminish from 12 h on (Fig. 5B).

Microbial stimulation has been shown to drive TLR4-mediated monocyte differentiation into mo-DC in immune T cell areas of lymph nodes.^16,18^ To determine if the CD11b^+^Bst-2^hi^ subset herein identified corresponded to mo-DC-like cells, we examined the surface expression of CD64 and DC-SIGN and compared it with that of other cell types, including pDCs and cDCs. These molecules were indeed reported to mark the mo-DCs generated by TLR agonist in tissues during inflammation.^16,18^ We found that, more than 93% of the CD11b^+^Bst-2^hi^ subset was CD64^+^, while more than 95% of cells remained CD64^−^ in the cDC and pDC fractions. In total, DCs with CD64^+^DC-SIGN^hi^ phenotype accounted for 40.6% of new emerging cells, and 1.87% and 1.69% of cDC and pDC, respectively (Fig. 5C). We further gated out splenic monocytes by CD11b and Ly6C expression, and found that reduction of CD11b^+^Ly6C^hi^ monocytes upon stimulation occurred (Fig. 5D) simultaneously with the increase of the CD11b^+^BST-2^hi^ population (Fig. 5A). Compared to steady-state monocytes, this population acquired DC morphology by elevating CD11c and MHCII expression levels (Fig. 5E). In TLR4^−/−^ mice, mobilisation of CD11b^+^BST-2^hi+^ DCs by LPSs was completely abolished, demonstrating a critical dependence on the TLR4 signalling pathway (Fig. 5F). Altogether these findings suggested that LPS stimulation triggered a TLR4-mediated emergence of CD11b^+^BST-2^hi^ DCs, recognised as mo-DC like cells, in the spleen. They also indicated that Bm-wt LPS played a limited but significant role in the mobilisation of such splenic mo-DC like cells while Bm*-wadC* LPS considerably favoured it.

### Bm-wadC LPS, but not Bm-wt LPS, induces TLR4-mediated phenotypic activation of splenic cDC subsets

**Figure 6.**
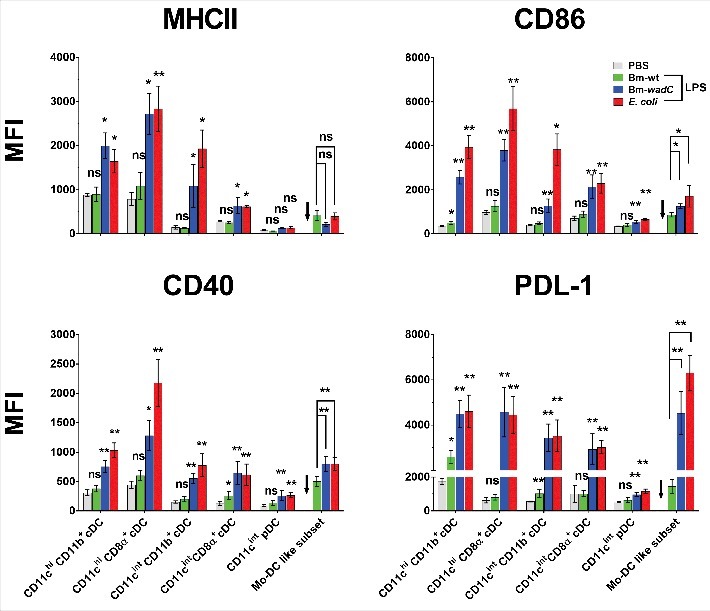
Bm-wt LPS poorly triggered phenotypic maturation of splenic DC subsets *in vivo*, in contrast to Bm-*wadC* LPS which promoted strong activation of DCs. 6–8 weeks-old C57BL/6J mice were injected intraperitoneally with 1xPBS (PBS), Bm-wt LPS (30 μg/mouse), Bm-*wadC* LPS (20 μg/mouse) or *E. coli* LPS (10 μg/mouse). 12 h later, mice were sacrificed, single-splenocyte suspensions were prepared and expression levels (MFI, Mean of Fluorescence Intensity) of MHCII, costimulatory molecules (CD86 and CD40) and inhibitory marker (PDL-1) on splenic DC subsets were determined by flow cytometry. Arrows point to the absence of data due to failure of mo-DC mobilisation by PBS. Data represent mean ± standard deviation of 3 independent experiments, each with n = 3 mice per condition. Statistical analysis was performed with the non-parametric one-way ANOVA test, followed by variance analysis with the Mann-Withney U test. Significant differences from PBS injected mice are shown; *, *P* < 0.05; **, *P* < 0.001. ns, non-significant.

**Figure 7.**
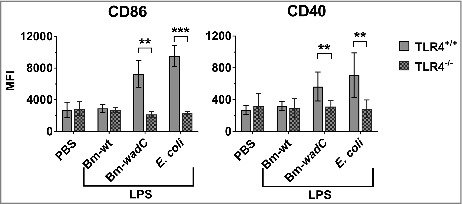
TLR4 was essential for LPS to drive maturation of splenic DCs. 6–8 weeks-old TLR4^+/+^ or TLR4^−/-^ mice were intraperitoneally injected with 1xPBS (PBS), Bm-wt LPS (30 μg/mouse), Bm-*wadC* LPS (20 μg/mouse) or *E. coli* LPS (10 μg/mouse). 12 h later, mice were sacrificed, single-splenocyte suspensions were prepared and expression levels (MFI, Mean of Fluorescence Intensity) of costimulatory molecules (CD86 and CD40) on total splenic CD11c^+^ cells were determined by flow cytometry. Data obtained from 3 experiments, each with n = 3 animals per condition, are shown. All error bars are standard deviations obtained from pooled data. Statistical analysis was performed with the non-parametric one-way ANOVA test, followed by variance analysis with the Mann-Withney U test. Significant differences from TLR4^+/+^ injected mice are presented; **, *P* < 0.01; ***, *P* < 0.001.

Finally, to characterise the maturation of splenic DC subsets upon Bm LPS stimulation *in vivo*, we examined the expression of a series of surface markers, indicators of activation (such as MHCII, CD86 or CD40) or inhibition (such as PDL-1). After 12 h of stimulation, Bm-*wadC* LPS dramatically induced the activation of cDC subsets (CD11b^+^ and CD8α^+^) from both CD11c^hi^ and CD11c^int^ populations at comparable levels to those observed with *E. coli* LPS in terms of activation markers (MHCII, CD86, CD40) and PDL-1. pDCs displayed a low activation status with a slightly increased expression of maturation markers (Fig. 6 and **S4)**. In comparison, Bm-wt LPS showed a poor ability to activate splenic DCs, with augmented CD86 expression in CD11c^hi^CD8α^+^cDCs and PDL-1 in CD11c^hi^CD11b^+^ and CD11c^int^CD11b^+^ cDCs only. In general, upon stimulation with Bm-*wadC* LPS or *E. coli* LPS, CD11c ^hi^ cDCs showed higher activation levels than CD11c^int^ cDCs. Despite the absence of mo-DC-like cells in PBS-treated mice, we could evaluate the activation of this DC subset upon LPS treatments. Bm-wt LPS elicited the lowest activation levels of costimulatory molecule expression in the mo-DC-like subset. Contrasting with the mild expression of activation markers, PDL-1 expression on these cells displayed a greater response to Bm-*wadC* LPS and *E. coli* LPS. In TLR4^−/−^ mice, splenic DCs failed to respond to any LPS leaving unchanged the basal levels of CD86 and CD40. These data demonstrated the absolute requirement of TLR4 for splenic DC maturation (Fig. 7).

### B. melitensis mutant with a partially defective LPS core structure displays enhanced cytokine secretion levels in vivo

**Figure 8.**
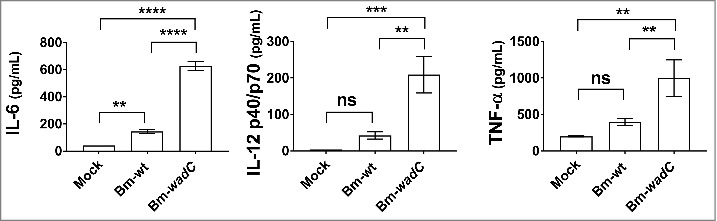
Bm-*wadC* mutant *Brucella* induced higher cytokine secretion than Bm-wt strain in infected mice. C57BL/6J mice were non-treated (Mock) or infected with *B. melitensis* 16M reference (Bm-wt) strain or Bm-*wadC* mutant strain for 8 days. Cytokine secretion was determined in sera by ELISA. Data obtained from 3 experiments, each with n = 3 animals per condition, are displayed. All error bars are standard deviations obtained from pooled data. Statistical analysis was performed with the parametric one-way ANOVA test, followed by variance analysis with the Tukey and Dunnett test. Significance was defined when P values were less than 0.05 (*, *P* < 0.05).

Our previous work demonstrated that the *wadC* mutants of both *B. abortus*^22^ and *B. melitensis*^23^ gave rise to an attenuated phenotype with lower CFUs in the spleen at 8 weeks post-infection *in vivo* as well as in GM-DCs 24 h post-infection *in vitro* compared to that observed with wild-type (WT) *Brucella*. In order to complete these studies and determine whether live *wadC* bacteria also impact the inflammatory response *in vivo*, we next investigated the effect of such *wadC B. melitensis* mutant on cytokine levels in the sera of infected mice. C57BL/6J mice were non-treated (Mock) or infected with Bm-wt or Bm-*wadC* strains for 8 days. Cytokine secretion was determined in sera by ELISA. Fig. 8 shows that *wadC* mutated *Brucella* triggered much higher levels of cytokine (IL-12p70, TNFα and IL-6) secretion in sera of mice at 8 days post-infection compared to those obtained with the WT strain. Altogether these data revealed that the *wadC Brucella* mutant drove a stronger inflammatory response than the WT strain.

## Discussion

Over the last decades *Brucella* LPS has been put forward as a poor immune stimulator that displayed less toxicity compared with other bacterial LPS, especially the *E. coli* one.^7,29–31^ Indeed, wild-type *Brucella* LPS did not induce inflammatory responses in macrophages and DCs, two of the most important sentinels of the immune system.^2,4,5,7^ This was attributed to its poor recognition by TLR4/MD-2, which is widely considered to be the major receptor complex for LPS binding and signalling.^22^ However, in recent years, a few reports demonstrated that *Brucella* LPS exhibited some adjuvant properties, which opened to debate its non-stimulatory immune properties.^32–34^ In addition, most of the findings at the cell level were obtained using macrophages and GM-DCs *in vitro*. In this context, further in-depth immunological characterisation of the effects of *Brucella* LPS in other cell models *in vitro* as well as *in vivo* appeared indispensable.

In this report, we first confirmed the inability of Bm-wt LPS to drive any activation of GM-DCs in terms of surface activation markers, inflammatory cytokine secretion, and T cell priming and proliferation *in vitro*. By comparing in this particular DC model the effects of a battery of LPSs that share some moeities with Bm-wt LPS, we found that LPSs made of a “*Brucella*-*type”* lipid A (*O. anthropi* 3331) or O-chain (*Y. enterocolitica* O:9) were in contrast able to drive strong GM-DC activation to levels comparable to those observed with *E. coli* LPS. Since the outer core deficiency (Bm-*wadC* LPS) by itself enhanced likewise GM-DC activation, including marked proliferation and activation of both CD4^+^ and CD8^+^ T cells, we identified this core component as the LPS molecular determinant responsible for *Brucella* poor immune cell activation ability. We propose that in *Brucella* wt LPS this oligosaccharide branching attached to the main oligosaccharide core section, which links lipid A and the O-polysaccharide, protrudes enough to mask lipid A determinants, hence hampering lipid A recognition by TLR4. Future crystallographic analyses of the *Brucella* wt LPS – TLR4 complex should determine the validity of this hypothesis. Collectively, these findings represent a substantial step in our understanding of the molecular mechanisms accounting for *Brucella* subversion of immune system recognition. Moreover, they define the LPS outer core deficiency as a key factor to be taken into consideration for the development of an efficient *Brucella* vaccine.

Towards this goal, we have previously published that both the *wadC* mutant of *B. abortus*^25,35^ and of *B. melitensis*^23^ give rise to an attenuated phenotype with reduced CFUs at 8 weeks post-infection compared to wild-type infected mice. We now show that compared to the wt strain, the *wadC* mutated *Brucella* from *B. melitensis* triggers much higher levels of pro-inflammatory cytokine secretion in sera of infected mice. Altogether these data indicate that the *wadC Brucella* mutant elicits a stronger inflammatory response than the wt strain resulting in an attenuated phenotype *in vivo* with a reduced bacterial burden. This enhanced immunoprotective ability opens up studies for assessing the potential of this mutant for vaccine development.

In addition, we reported that our murine GM-DC data could be translated to human GM/IL-4 mo-DCs, i.e. Bm-*wadC* LPS also stimulated cytokine secretion in human cells unlike Bm-wt LPS. These findings revealed that *Brucella* has developed some “universal” mechanisms of immune response subversion (including the inhibitory ability of its oligosaccharide LPS core) which operate both in human and mouse cells, although the latter animal recipient is not a natural *Brucella* host.

Our comparative approach also led us to disclose that Bm-*wadC* and *O. anthropi* 3331 LPSs, which share a “*Brucella* type”-lipid A, drove intermediate IL-10 secretion levels, whereas *E. coli* and *Y. enterocolitica* O:9 LPSs, which bear the same “*E. coli* type-lipid A” elicited 5 times more in GM-DCs. These results unravelled the “*E. coli* type”-lipid A as a critical feature for massive secretion of a key anti-inflammatory cytokine. As IL-10 has been shown to be one of the crucial cytokines involved in endotoxic shock driven by *E. coli* LPS,^36^ these data raise the question of the specific contribution of “*E. coli* type-lipid A” moiety in the etiology of this multiorgan failure syndrome upon infection. Concerning *Brucella*, further investigation will determine if its peculiar lipid A component is required to mediate mild IL-10 secretion *in vivo*. Moreover, assessing whether endotoxic shock might be generated by artificially augmented IL-10 to serum levels alike those produced by *E. coli* LPS in Bm-wt LPS injected mice, would formally shed light on one of the potential reasons explaining the low endotoxicity of *Brucella* LPS.

We extended our investigation of *Brucella* LPS immunomodulatory properties from GM-DCs to another DC system, the FL-DCs. We demonstrated that according to the DC model used, Bm-wt LPS differed in its ability to trigger immune cell activation. FL-DC pDCs displayed almost no activation status with only slightly increased expression of maturation markers, probably due to by-stander effects caused by the other DC subsets. The absence of pDC activation by any LPS, in FL-DCs *in vitro* or in splenic pDCs *in vivo*, most likely results from the low expression levels of TLR4 in these cells compared to that of other murine DC populations.^37^ In contrast, Bm-wt LPS efficiently promoted maturation of CD11b^+^ and CD24^+^ FL-cDC subsets by elevating MHCII, costimulatory molecules and by significantly releasing Th1 cytokines as well as low levels of IL-10. The differential response of the two DC models to a given microbial stimulation might result from their distinct intrinsic activation thresholds. In FL-DCs, the poor and non-significant secretion of IL-12p70 and IFN-γ by Bm-wt LPS may account for the partial activation (i.e. poor inducing T cell proliferation capacity) of these cells. The inability to stimulate secretion at high levels of such Th1 inducers by Bm-wt LPS in the FL-DCs *in vitro* seems to reflect the situation occuring in splenic DCs *in vivo*. Indeed, only the Bm-*wadC* LPS, but not the Bm-wt LPS, triggered significant maturation (i.e. up-regulation of co-stimulatory molecules) of splenic cDC subsets. Incomplete activation of DCs might contribute to the subversion of the immune system by *Brucella* LPS and participate in the establishment of *Brucella* chronicity, as IFN-γ and IL-12 have been shown to control *Brucella* infection.^38–42^ It remains that our work challenged the accepted paradigm by proving that *in vitro Brucella* LPS might exert some stimulatory effects on certain DC subsets. As in GM-DCs, the *wadC* defect relieved the inhibitory effect of *Brucella* LPS core structure on activation of FL-DC subsets, by further increasing maturation marker expression and cytokine release. These results therefore confirmed in another murine *in vitro* system the masking ability of *Brucella* core branching structure on lipidA immunogenicity. *Y. enterolitica* O:9 LPS-induced IL-10 levels were the highest in FL-DCs, but in the range of those engendered by the other LPS, reflecting a specific response of the FL-cDC subsets compared to that of GM-DCs. As regards Th1 cytokine secretion (IL-12p40, IL-12p70, TNF-α, IL-6, and IL-1β) in FL-DCs, *O. anthropi* 3331 was always the strongest inducer, revealing a peculiar role for *O. anthropi* 3331 core and oligosaccharide moieties.

The spleen is an ideal reservoir for a diversity of myeloid and dendritic cells distinct in antigen presenting functions.^9^ When examining the modulatory effects of *Brucella* LPS on DCs *in vivo*, we found that only the Bm-*wadC* LPS, but not the Bm-wt LPS, triggered significant maturation, i.e. notablly increased expression of surface activation markers, of splenic cDC subsets. The CD11c^hi^CD11b^+^and CD11c^int^CD11b^+^ cDCs on the one hand and the CD11c^hi^CD8α^+^ cDCs on the other hand were the sole splenic DC subsets which exhibited a faint up-regulation by *Brucella* wt-LPS of PDL-1 and CD40, respectively. CD8α^+^ cDCs are the predominant producers of IL-12, important for CD8^+^ T cell proliferation.^43^ CD8α^−^ cDCs have a weaker cross-priming ability; upon LPS stimulation, they are relocalised from the marginal zone of spleen to T cell areas and secrete inflammatory chemokines. However, their primary role still remains unclear.^44^ The poor activation of these cDC subsets by *Brucella* wt-LPS *in vivo* most likely participates in the limited inflammatory response occurring during the course of *Brucella* infection.

We also observed that both *Brucella* LPS and *E. coli* LPS induced in a TLR4-dependent manner, concomitantly with the disappearance of splenic monocytes, the emergence of a cell type with mo-DC like phenotypic features. Mo-DCs are efficient antigen-presenting cells and good inducers of inflammatory responses.^14,15,45^ The weak, but significant, mobilisation of splenic mo-DC like cells elicited by Bm-wt LPS may, similarly to cDCs, restrict the *in vivo* inflammation during infection and promote setting up of *Brucella* replicative niche. This hypothesis is consistent with our previous *in vitro* report^24^ showing that higher DC activation correlates with an inefficient ER replicative niche targeting of intracellular bacteria.

Collectively, these findings illustrate how a poor activation of DC subsets *in vivo* may contribute to the immune system avoidance elicited by *Brucella*, ultimately causing establishment of chronic disease.

In conclusion, this study extensively documented both *in vitro* and *in vivo Brucella* LPS immunomodulatory abilities, shedding light on its effect on cDC subsets and mo-DC like cells in particular. The enhanced stimulatory property of *Brucella* LPS with a core structure defect pleads for the assessment of the benefits of this mutation for new vaccine strategies targeting bacterial LPS.

## Materials and methods

### Ethics statement

Animal experimentation was conducted in strict compliance with good animal practice as defined by the French animal welfare bodies (Law 87–848 dated 19 October 1987 modified by Decree 2001–464 and Decree 2001–131 relative to European Convention, EEC Directive 86/609). All animal work was approved by the Direction Départementale des Services Vétérinaires des Bouches du Rhône. Live *Brucella* infection protocol was approved by the ANSM. All efforts were made to minimise suffering during animal handling and experimentation. As regards human PBMCs, blood was taken from healthy volunteers acquired under protocols approved by the Institutional Review Board (IRB) of Baylor Research Institute (BRI).

### Mice

6–8 week-old female C57BL/6J mice from Charles River, TLR4^−/−^ mice^46^ on a C57BL/6J background and OT-I, OT-II TCR transgenic mice on a C57BL/6J background from the Jackson laboratory were used. Animals were housed in cages with water and food ad libitum in the CIML or CIPHE animal house facilities (for the latter, under biosafety containment conditions for 2 weeks before the start and all along infection with live bacteria).

### Lipopolysaccharides

LPS from different *Brucella* strains (16M Biovar 1 reference strain, wt or *wadC* mutant^23^), *E. coli* ATCC 35218, *Y. enterocolitica* O:9 (MY79), *O. anthropi* (LMG 3331T) were extracted and purified as described previously.^22,27^ Briefly, *Brucella* and *Y. enterocolitica* LPSs were obtained from the phenol phase of a water-phenol extract. These crude LPS preparations were then purified by removing the free-lipids by solvent extraction, digestion with nucleases and proteinase K and ultracentrifugation.^27^ *O. anthropi* LPS (obtained from the water phase) was purified in the same way as *Brucella* LPS. *E. coli* LPS was obtained from the water phase of a similar phenol-water extract and then purified by the phenol-water deoxycholate method.^47^ A stock of 1 mg/mL of each LPS was prepared in pyrogen free sterile water, sonicated briefly and sterilized by autoclaving. Prior to use, the stock was heated at 56°C for 15 min and then cooled to room temperature. The molar mass ratio of different LPS (*E. coli* LPS: Bm-wt LPS: Bm-*wadC* = 1:3:2) was calculated based on the KDO content, as the use of this LPS core marker allows for correction of differences in dry weight that could result from different O-polysaccharide lenghts and lipid A molecular weights.^48^ To compare the immunomodulatory properties of different LPSs *in vivo*, equal molarity of LPSs were intraperitoneally injected in mice, with a dosage of 10 µg, 30 µg or 20 µg per mouse for *E. coli* LPS, Bm-wt LPS and Bm-*wadC*, respectively.

### In vitro generation of BMDCs

BMDCs were prepared from 6–10 week-old C57BL/6J female femurs and tibias as previously described.^24^ Briefly, bone ends were cut off and bone marrow was flushed with RPMI medium (GIBCO, 21875–034) containing 5% FCS and 50 mM of 2-mercaptoethanol (GIBCO, 31350–010). Red blood cells were removed by 1 min exposure to 1xRBC lysis buffer solution (eBioscience, 00–4333–57). For GM-DCs, 3 × 10^6^ cells were seeded onto 6-well plates in 5 mL medium containing 0.8 % supernatant of the J558L GM-CSF producing cell line. Medium was changed at day 2.5 and GM-DCs were ready to use at day 5. For FL-DCs, 8 × 10^6^ cells were seeded onto 6-well plates in 4 mL medium containing 3% supernatant of the B16 FLT3 Ligand producing cell line. FL-DCs were ready to use at day 8.5–9 without any change of medium.

### Stimulation of BMDCs

Cells were mock-treated or stimulated for 24h with Bm-wt LPS (10 µg/mL), Bm-*wadC* LPS (10 µg/mL), *E. coli* LPS (100 ng/mL). The dosage of LPS for stimulation was optimised previously^22^; in the GM-DC model, saturating concentration for Bm-*wadC* LPS was 10  μg/mL, while that of *E. coli* LPS was 100 ng/mL. Cells were collected for FACS analysis and supernatant was kept at −80°C. Cytokines were measured by ELISA kits for IL12p70 (88–7121-76), IL-12/IL-23 total p40 (88–7120-86), IL-6 (88–7064-88), IL-10 (88–7105-88), IFN-γ (88–7314-76), TNF-α (88–7324-88), IL-1β (88–7013-86).

### Human mo-DCs 

Human monocyte-derived DCs were generated from Ficoll-separated PBMC from healthy volunteers. Monocytes were enriched from the leukopheresis according to cellular density and elutriation following manufacturer's instructions. For DC generation, monocytes were resuspended in serum-free Cellgro DC culture supplemented with 1ng/mL GM-CSF and 0.1 ng/mL IL-4 and kept in culture for 7 days. The mo-DCs were then stimulated as previously described with each LPS at 20 ng/mL.^49^

### Flow cytometry

Cells were harvested and stained for 20 min at 4°C with the antibody mix. After a wash in PBS with 2% of FCS, cells were stained with Fixable Viability Dye eFluor 506 (eBiosciences, 65–0866-14) for 10 min at room temperature. Cells were then fixed for 20 min in 3.2% PFA at room temperature. mAbs in the staining mix for BMDCs or splenocytes were the following: CD11c PeCy7 (N418), MHC II PE (M5/11.15.2), CD86 FITC (GL-1), CD86 PE (GL-1), CD80 PeCy5 (16–10A1), CD40 APC (HM40–3), CD24 FITC (M1/69), NK1.1 APC-Cy7 (PK136), PDL-1 BV605 (10F.9G2) from Biolegend; MHCII A700 (M5/11.15.2), CD11b eF450 (M1/70), Bst-2 EF610 (eBio927), DC-SIGN APC (eBio22D1) from eBiosciences; CD40 PeCy5 (3/23), B220 PETXR (RA3–6B2), CD3 APC-Cy7 (145–2C11), CD19 APC-Cy7 (1D3), CD64 PE (X54–5/7.1), CD8α BV711 (53–6.7) from BD Biosciences. Flow cytometry was performed using a FACS LSRII-UV (BD Biosciences) and data were analysed with the FlowJo_v9.9.4 software.

### Murine splenocyte preparation

Mice were anesthetised by CO_2_ inhalation. Spleens were dissected from abdominal cavity, digested for 20 min at 37°C with type II collagenase (Worthington Biochemical Corporation, LS004174) and DNase I (Sigma, DN25–100MG) and then treated with 10 mM EDTA to stop digestion. Pieces of spleen cut by scissors were crushed with a syringe plunger in a 70-μm nylon strainer cell strainer and filtered in PEF buffer (1xPBS, 5 mM EDTA, 2% FCS). Red cell lysis buffer was used to remove red cells. Single splenic cell suspensions then proceeded to FACS analysis.

### In vitro antigen presentation assays

GM- or FL-DCs (10^4^ cells) were incubated for 16 h in 96-well culture plates with endotoxin-free OVA at 500 μg/mL (Hyglos, 321001). They were then stimulated or not (Mock) for 8 h with different LPSs as described above and washed. OT-I and OT-II T cells were isolated from spleens of OT-I or OT-II mice using a CD8^+^ or CD4^+^ T cell-negative isolation kit (Dynal, Invitrogen, 11348D and 11352D), respectively. Purity was determined by staining with CD4, CD8 and CD5. Purified OT-I and OT-II T cells were finally resuspended in 1xPBS containing 10 mM CFSE (Molecular Probes, C34554) for 10 min at 37°C. CFSE-labelled OT-II or OT-I cells (10^5^ cells) were added to the BMDCs prepared as aforementioned. The proliferation of OT-I and OT-II T cells was assessed by measuring CFSE profiles by flow cytometry after 48 h of co-culture.

### Splenic DC stimulation in vivo

4 h, 12 h, 24 h or 48 h after intraperitoneal injection of mice with 1xPBS or LPS, spleens were collected, single-cell suspensions were prepared by gentle dissociation and enzymatic digestion as described above and then analysed by flow cytometry.

### In vivo Brucella infection

C57BL/6J mice were non-treated (Mock) or infected with *B. melitensis* 16M reference (Bm-wt) strain or Bm-*wadC* mutant strain. After 8 days, sera were collected and kept at −80°C until cytokine measurement.

### Statistical analysis

All values are expressed as mean ± standard deviation. All experiments were performed at least three times in triplicate otherwise indicated. Statistical analyses were done using with the GraphPad Prism software. Non-parametric one-way ANOVA test followed by variance analysis with the Benjamin and Hochberg test were performed, otherwise indicated in the figure legend. Differences between values were considered significant at *P* < 0.05 (*, *P* < 0.05; **, *P* < 0.001; ***, *P* < 0.0001; ****, *P* < 0.00001).

## Abbreviations

Bm: Brucella melitensis
DC: dendritic cells
FL-DC: Flt3L-derived DCs
Flt3L: Fms-like tyrosine kinase 3 Ligand
GMCSF: Granulocyte macrophage-colony stimulating factor
GM-DC: GM-CSF-derived dendritic cells
IFN: interferon
IL: interleukine
LPS: Lipopolysaccharide
mo: monocyte
PBMC: peripheral blood mononuclear cells
TLR: toll-like receptor
TNF: Tumor necrosis factor
