# Supplementary material for: Immunomodulatory properties of Brucella melitensis lipopolysaccharide determinants on mouse dendritic cells in vitro and in vivo
Source: Virulence. 2017 Oct 2;9(1):465–79. doi: 10.1080/21505594.2017.1386831 (PMC5955181; doi:10.1080/21505594.2017.1386831)
Supplement: KVIR_S_1386831.docx [file kvir-09-01-1386831-s001.docx]

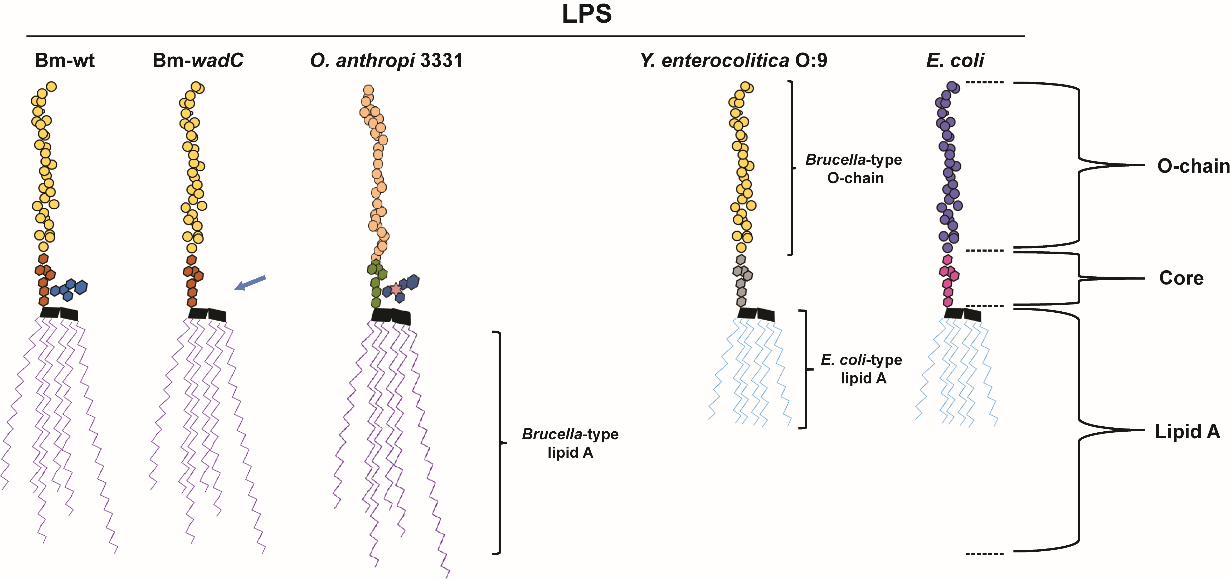


**Figure S1.  Diagram of the different LPSs used in this study.** The LPS of gram-negative bacteria consists of three consecutively linked sections: lipid A, core oligosaccharide and O-chain. Prototypical LPS, the *E. coli* one, differs by its overall structure from that of *Brucella melitensis* (Bm-wt LPS). *Brucella*-type lipid A is made of a diaminoglucose disaccharide substituted with C16, C18, C28 and other very long acyl chains, in contrast to *E. coli*-type lipid A formed by a glucosamine disaccharide joined predominantly to C12 to C14 acyl chains in ester, amide and acyl-oxyacyl bonds. Bm-*wadC* LPS exhibits a partially defective oligosaccharide core, with a branching missing (indicated by an arrow), but intact O-polysaccharide and lipid A compared to Bm-wt LPS. *Ochrobactrum anthropi* 3331 LPS carries a *Brucella*-type lipid A but different core and O-chain sugars. *Yersinia enterocolitica* O:9 LPS displays the same O-chain homopolymer as Bm-wt LPS but its lipid A is alike *E. coli* LPS.


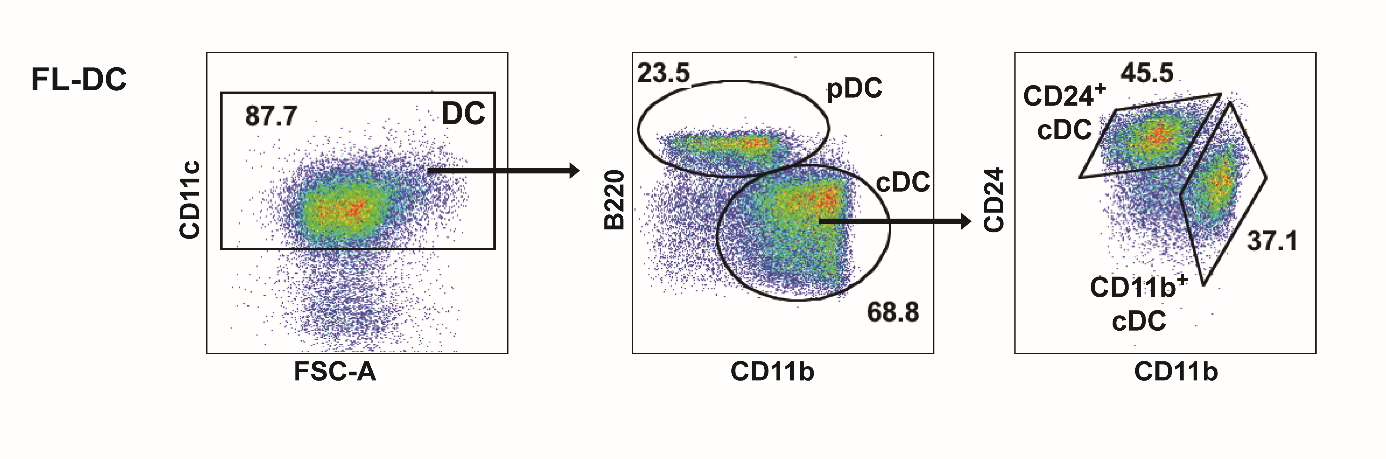


**Figure S2. Identification of bone marrow derived FL-DC subsets *in vitro*.**

FL-DCs were prepared by culturing freshly isolated bone marrow cells *in vitro* for 9 days in presence of Flt3-ligand, and their phenotype was assessed by flow cytometry. FL-DCs were distinguished into 3 populations (pDC, CD11b^+^cDC and CD24^+^cDC) with the indicated combinations of surface markers.


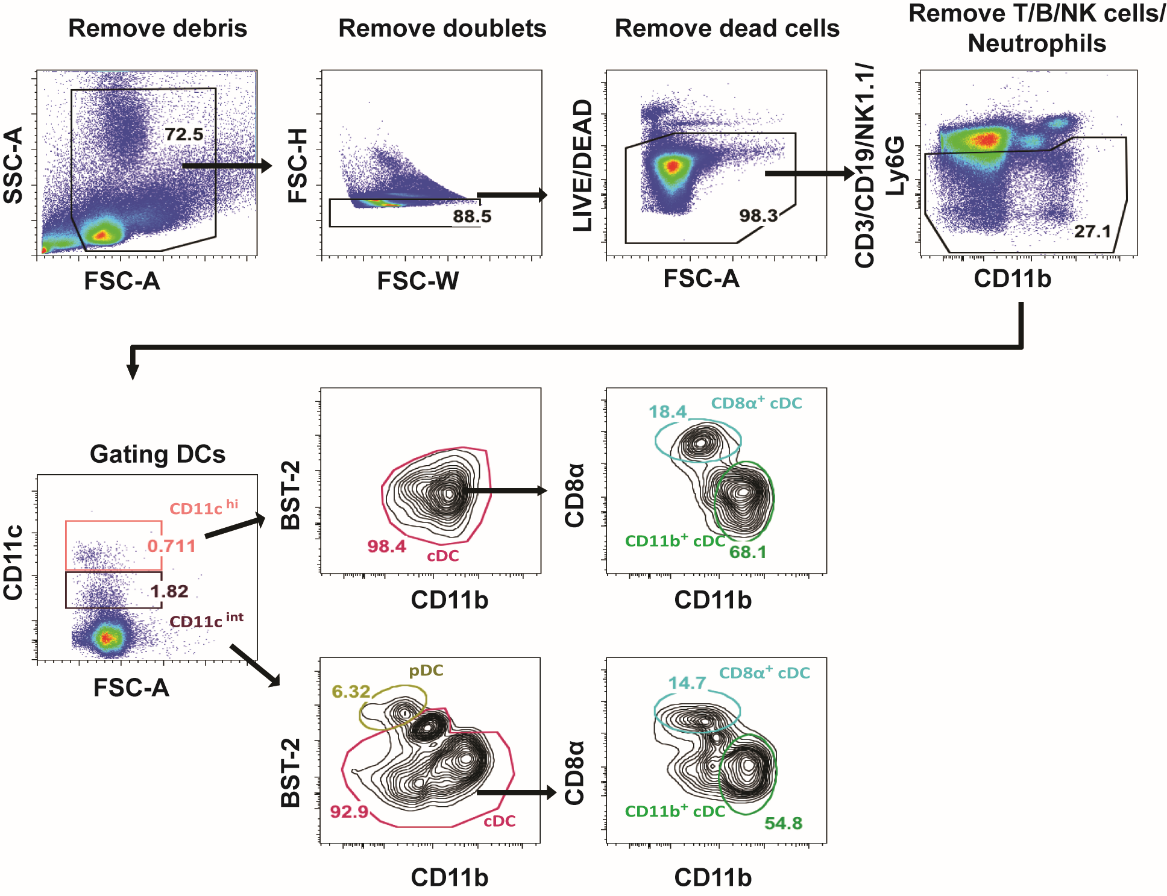


**Figure S3. Characterisation of splenic DCs present at steady-state.**

Single-cell suspensions from spleens were prepared and analysed by flow cytometry. After excluding T cells, B cells, NK cells and neutrophils, the remaining was analysed for CD11c expression and DCs were divided into two populations: CD11c^hi^ DCs and CD11c^int^ DCs. CD11c^hi^ cells comprised CD8a^+^ conventional DC (CD8α^+^cDC) and CD11b^+^ conventional DC (CD11b^+^cDC). Steady-state CD11c^int^ DCs were composed of pDC and cDCs, distinguished by BST-2 and CD11b expression, and cDCs were subdivided into CD8α^+^ and CD11b^+^ subsets.

**
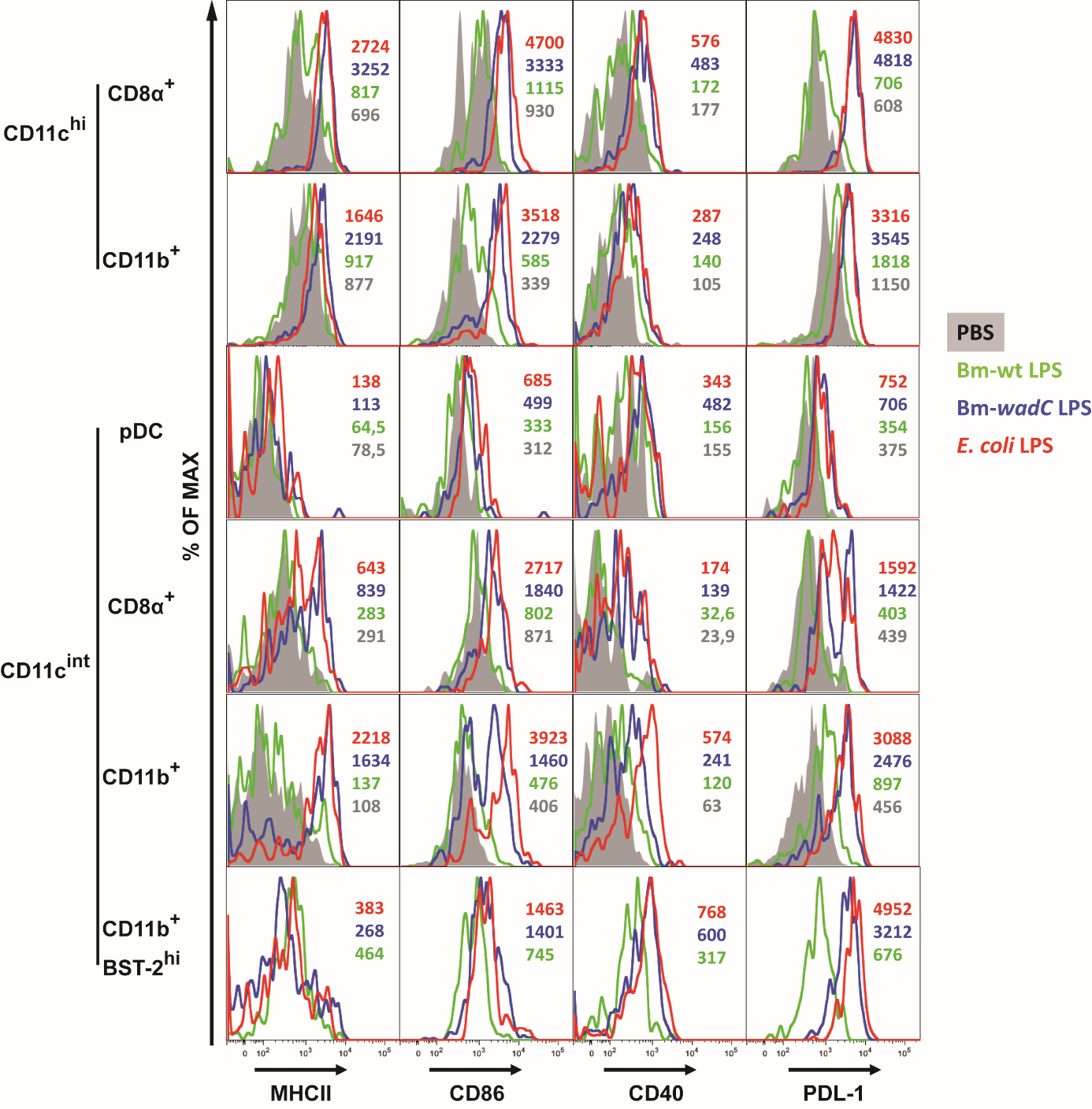
**

**Figure S4. Bm-wt LPS failed to induce any phenotypic maturation of splenic DCs subsets *in vivo* in contrast to *wadC* LPS, which promoted high expression of activation & inhibitory markers on several DC subsets similarly to *E. coli* LPS.**

Single-cell suspensions from spleens were prepared as described in Figure S3 and analysed by flow cytometry for expression of MHCII, CD86, CD40, PDL-1 on the various splenic DC subsets.
